# Prognostic importance of expression of mini-chromosome maintenance proteins (MCMs) in patients with nasopharyngeal cancer treated with curative radiotherapy

**DOI:** 10.1016/j.bjorl.2021.05.012

**Published:** 2021-06-08

**Authors:** Gul Kanyilmaz, Pembe Oltulu, Berrin Benli Yavuz, Meryem Aktan

**Affiliations:** a Necmettin Erbakan University, Meram Faculty of Medicine, Department of Radiation Oncology, Konya, Turkey; b Necmettin Erbakan University, Meram Faculty of Medicine, Department of Pathology, Konya, Turkey

**Keywords:** Minichromosome maintenance protein (MCM), Nasopharyngeal cancer, Radiotherapy, Survival outcomes

## Abstract

**Objective:**

The prognostic importance of minichromosome maintenance complex expression in nasopharyngeal cancer is still unknown. We aimed to find whether minichromosome maintenance complex 2–7 expression may potentially be used to predict the prognosis of nasopharyngeal cancer patients treated with definitive radiotherapy.

**Methods:**

Between April 2007 and July 2020, patients with nasopharyngeal cancer treated with radiotherapy were identified. Immunohistochemical analysis was performed on formalin-fixed paraffin-embedded tissues of cases. A single pathologist analyzed the histologic specimens of all patients.

**Results:**

Totally, 67 patients were included. The median followup was 75.3 months. Higher tumor (T) stage was correlated with minichromosome maintenance complex 2 overexpression. Minichromosome maintenance complex s expression was also associated with histopathologic subgroups. According to univariate analysis, AJCC stage, histopathological subgroups, tumor response after treatment, minichromosome maintenance complex 2, 3, 5, 6 and 7 expression were the prognostic factors that predict overall survival. According to multivariate analysis minichromosome maintenance complex 7 expression was the only prognostic marker for both progression-free survival and overall survival.

**Conclusion:**

The overexpression of minichromosome maintenance complex 2, 3, 5, 6 and 7 indicated bad prognosis. Minichromosome maintenance complex 7 was an independent prognostic factor for survival outcomes in nasopharyngeal cancer and may be a potential therapeutic target for treatment.

## Highlights

• In nasopharyngeal cancers, the most important therapeutic problem in treatment resistance is radiation resistance.
• Minichromosome maintenance complex has a critical function in DNA replication and oncogenic signaling pathways.
• In the current study, the overexpression of minichromosome maintenance complex 2, 3, 5, 6 and 7 indicated bad prognosis.
• Minichromosome maintenance complex 7 was an independent prognostic factor for survival outcomes and may be a potential therapeutic target for the treatment of nasopharyngeal cancer.

## Introduction

Nasopharyngeal cancer originates from the nasopharyngeal epithelium.^1^ It is less common than other types of cancer and the incidence shows a regional distribution.^2^ According to GLOBOCAN data, 129,079 people were diagnosed with nasopharyngeal cancer in 2018 and 72,987 people died with this diagnosis.^2^ Most of the patients are diagnosed with local advanced stage; the primary treatment option for nasopharyngeal cancer is radiotherapy (RT) owing to the anatomical position of the nasopharynx, as it is not suitable for surgery.^1^, ^3^ RT is applied alone in stage I disease.^1^ Although there are some studies showing the benefit of concurrent chemotherapy (ChT) applications with RT in stage II disease, there is no randomized study investigating this issue.^1^ Since the end of the 1900s, RT and ChT have been applied together in stage III and IV disease. With combined treatment, a 30% increase in 3 year overall survival (OS) was found at these stages.^4^ A positive effect of adjuvant ChT on OS has not been demonstrated.^5^ Despite aggressive treatment approaches, the results are still not satisfactory.

The most important therapeutic problem in treatment resistance is radiation resistance,^6^, ^7^ which includes mechanisms such as hypoxia,^8^, ^9^ cell cycle stage,^10^, ^11^ DNA damage and repair mechanisms,^12^, ^13^ apoptosis,^14^, ^15^ oncogenes and growth factors,^16^, ^17^ cancer stem cells and epigenetic modifications in genes.^18^

Minichromosome maintenance (MCM) protein family takes an active role in the initiation phase of eukaryotic DNA replication process. It is not surprising that MCM overexpression is seen in many cancers, when the critical role of MCM on DNA replication is considered.^19^ Studies have shown that MCM complex subunits (MCM2–7) play an active role in cell proliferation, invasion and metastasis.^19^, ^20^ However, the results of the studies that investigate the effect of MCM complex on survival are contradictory.^19^, ^20^ Although there are some studies reporting a decrease in survival rates with increasing MCM complex subunits in gastric, ovarian, breast, gliomas, bronchoalveolar lung cancer, gallbladder, osteosarcoma and urinary cancers, there are also studies that reported a better prognosis with increased MCM complex subunits in lung adenocarcinoma, colorectal cancers and ER (+) breast cancer.^19^, ^20^

On the other hand, the prognostic importance of MCM expression in nasopharyngeal cancer is still unknown. Because of the MCM2–7 complex has a critical function in DNA replication and oncogenic signaling pathways, we aimed to study whether MCM2–7 expression may potentially be used to predict the prognosis of patients with nasopharyngeal cancer treated with definitive radiotherapy as well as potential therapeutic targets for anticancer treatments.

## Methods

### Patients’ characteristics

Ethical approval of this study was obtained from the committee of our university. The approval number of the ethics committee was 2018/1579. Between April 2007 and July 2020, patients with nasopharyngeal cancer treated with definitive radio- (-chemo) therapy were identified. Among them, patients whose pathological diagnosis was performed in our hospital were included in the current research.

All patients were biopsy-proven, newly diagnosed nasopharyngeal cancer. The excluding criteria of the study were: followup time < 6 months, age < 18 years, Karnofsky performance status (KPS) < 70, history of prior head and neck radiotherapy, a history of other type of cancer within the last 5-years and finding of metastasis at the time of diagnosis.

### Treatment

#### Radiotherapy

All patients were immobilized with thermoplastic head and shoulder mask in a supine position. Intravenous contrast enhanced computed tomography (CT) simulation was carried out at 3 mm slice thickness. Planning CT was fusioned with magnetic resonance imaging (MRI) and/or positron emission tomography (PET-CT) images for all patients. Treatment volumes were delineated slice-by-slice on the planning CT scans by using an individual protocol in compliance with International Commission on Radiation Units and Measurements reports 62 and 83. The gross tumor volume (GTV; primary tumor and metastatic lymph nodes) plus margins received 70 Gy/35 fractions/5 fraction per week. The clinical target volume (CTV; clinically uninvolved areas at risk for recurrence) plus margins received 54–63 Gy/35 fractions/5 fraction per week. Eclipse Treatment Planning System version 8.9.08 (Varian, Palo, Alto, CA) was utilized for treatment planning. Intensity modulated radiation therapy (IMRT) with simultaneous integrated boost technique was used for the treatment of patients.

### Chemotherapy

Concurrent chemotherapy with or without sequential chemotherapy (neoadjuvant or adjuvant) was administered to all patients except stage I disease. The chemotherapy regimen was generally applied as in accordance with the clinical practice guidelines.

### Followup

During the treatment, all patients were examined at least once in a week. The patients were followed up every 2–3 months for the first two years, every four months for the third year, every six months and once a year for the fourth and fifth years after the completion of therapy. At each followup visit, a historical and clinical examination, including flexible endoscopy, was done. MRI of the head and neck were performed annually for the first five years. Additional examinations were added as needed to assess local or distant recurrence.

### Immunohistochemistry (IHC)

Immunohistochemical analysis was performed on formalin-fixed paraffin-embedded tissues of cases diagnosed previously as nasopharyngeal cancer. Archival slides stained with hematoxylin and eosin were examined firstly. Paraffin blocks compatible with slides containing sufficient amount of tumoral tissue for evaluation were collected from the archive. For immunohistochemical analysis, 4 μm-thick sections of all the blocks were cut with the microtome, placed on positively charged slides, deparaffinized with xylene and dehydrated with ethanol solutions. Immunostaining for MCM2 (Clone E-8, mouse monoclonal, Santa Cruz Biotechnology, Inc., Dallas, TX), MCM3 (Clone E-8, mouse monoclonal, Santa Cruz Biotechnology, MCM4 (Clone G-7, mouse monoclonal, Santa Cruz Biotechnology, Inc., Dallas, TX), MCM5 (Clone E-10, mouse monoclonal, Santa Cruz Biotechnology, Inc., Dallas, TX), MCM6 (Clone H-8, mouse monoclonal, Santa Cruz Biotechnology, Inc., Dallas, TX), MCM7 (Clone 141.2, mouse monoclonal, Santa Cruz Biotechnology, Inc., Dallas, TX) was performed in all cases using Ventena Benchmark XT IHC autostainer system.

### Evaluation of immunohistochemical staining

**Figure 1:**
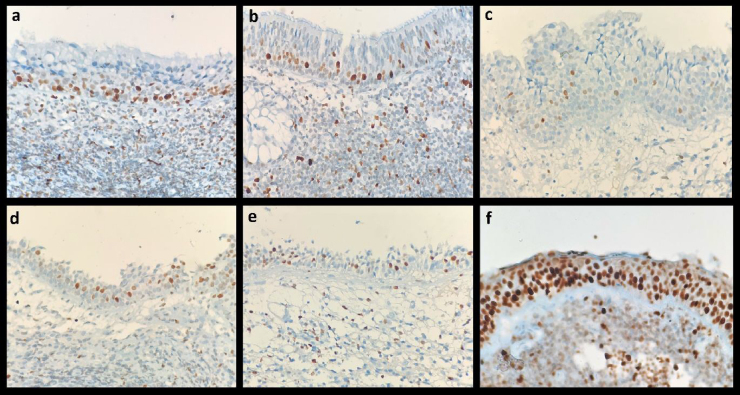
Rare brown MCM staining in basal nuclei in normal nasopharyngeal epithelium: (a) MCM2; (b) MCM3; (c) MCM5; (d) MCM6; (e) MCM7; (f) MCM6 strong staining in epithelium with random atypical dysplasic changes in one of the samples. Magnification ×400.

**Figure 2:**
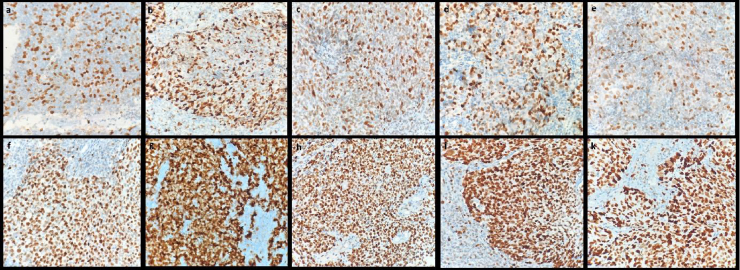
Brown positivity rates in nuclei of tumor cells: (a) low-MCM2; (b) low-MCM3; (c) low-MCM5; (d) low-MCM6; (e) low-MCM7; (f) high-MCM2; (g) high-MCM3; (h) high-MCM5; (i) high-MCM6; (k) high-MCM7 in MCM immunohistochemical staining. Magnification ×400.

A single histopathologist analyzed the histologic specimens of all patients. Positive immunoreactions of MCMs were detected by the brown color of chromogen in a pale blue background on all immunohistochemically stained slides. Immunoreactive, brown stained, nuclei were calculated among at least 500 tumor cells separately at ×400 magnification with a conventional light microscope (Olympus, BX41). MCMs staining in basal nuclei in normal nasopharyngeal epithelium are shown in Fig. 1. Nuclei labeling index (LI) for each case was determined as the percentage of nuclei with a positive reaction. The specimens with the low and high intensity of the nuclei staining are presented in Fig. 2.

### Statistical analysis

Statistical Package for Social Sciences for Windows version 13 (SPSS. Inc. Chicago. IL) was carried out for all statistical analyses. The overall survival (OS) was calculated by the date of pathological diagnosis until the date of last follow-up or until the death. The progression-free survival (PFS) was calculated by the date of pathological diagnosis until the date of the first observation of locoregional recurrence or distant metastasis or death because of nasopharyngeal cancer. OS and PFS were measured by Kaplan–Meier analysis and the two-sided long rank test was utilized to compare the survival curves of subgroups. The possible associations between variables and survival outcomes were evaluated by Cox regression analysis. Variables with statistical significance in univariate analysis were inputted as covariates in multivariate regression analysis. The descriptive statistics were performed to measure the mean value, the median value, the proportion value, the range and the standard deviation values. Pearson’s Chi-Square test was used to compare the categorical variables. Independent sample *t*-test and ANOVA test was utilized to compare the continuous variables. ROC (receiver operating characteristics) curve analysis was used to calculate the ability of MCM value for the prediction of disease recurrence, distant metastases or death. A two-sided p-value <0.05 was accepted as statistically significant.

## Results

### Patients characteristics and follow-up

Totally, 67 patients with nasopharyngeal cancer were included in the current study. The median followup was 39.7 (range 6.36–130.82) months. The patient, tumor and treatment characteristics are demonstrated in Table 1.

**Table 1:** Patients, tumor and treatment characteristics.

Variables	Nº of patients (total = 67)	%
Age (years)		
Median	51	
Range	19–81	73
Sex		
Male	49	
Female	18	27
Clinical stage^a^		
Stage I	6	9
Stage II	5	8
Stage III	31	46
Stage IV	25	37
Stage classification		
Stage I–III	42	63
Stage IV	25	37
Histology		
Keratinizing	16	24
Nonkeratinizing	48	71
Basaloid	3	5
Concurrent chemotherapy		
Yes	61	91
No	6	9
Sequential chemotherapy		
Yes	18	27
No	49	73
MCM2 value		
Low (<81)	21	31
High (≥81)	46	68
MCM3 value		
Low (<99)	40	60
High (≥99)	27	40
MCM5 value		
Low (<92)	39	58
High (≥92)	28	42
MCM6 value		
Low (<94)	30	45
High (≥94)	37	55
MCM7 value		
High (<88)	42	63
Low (≥88)	25	37

MCM, Mini Chromoso Memaintenance.

a According to version of American Joint Committee on Cancer (AJCC) 8th edition, 2017.

### MCMs evaluations

We found that MCM2 expression ranged from 18% to 100% (median; 90%), MCM3 expression ranged from 30% to 100% (median: 98%); MCM5 expression ranged from 25% to 100% (median: 86%), MCM6 expression ranged from 28% to 100% (median; 95%) and MCM7 expression ranged from 16% to 100% (median: 85%). We couldn’t demonstrate the MCM-4 expression levels because of some technical problems that may have been caused by the transport systems of MCM4 antibody that need to protect the cold chain or its production stages, and thus we were unable to see any reaction in none of the patients. ROC analysis was done separately to determine the optimal cut-off point for each MCM expressions with continous variable value. According to ROC curve, the optimal cut-off point was 81% for MCM2, 99% for MCM3, 92% for MCM5, 94% for MCM6 and 88% for MCM7. Below these cut-off values are considered as low levels (MCM-low), while above these values are considered as high levels (MCM-high).

### Association between MCMs expression and clinicopathologic variables

The relationship between the clinicopathological variables and MCMs expression was also examined. The results demonstrated that higher tumor (T) stage was correlated with only MCM2 overexpression (Table 2); (*p* = 0.02). There was not any association between AJCC stage and the other MCMs expression. Furthermore, the results showed that MCMs expression was associated with histopathologic subgroups. The relationship between histopathologic subgroups and MCM expression are presented in Table 3.

**Table 2:** The relationship between MCM2 expression and T stage.

	T1 stage	T2 stage	T3 stage	T4 stage	*p*
**MCM2 value**					
Low	12 (55%)	3 (13%)	2 (30%)	4 (27%)	0.02^a^
High	10 (45%)	20 (87%)	5 (70%)	11 (73%)	
Total	22 (100%)	23 (100%)	7 (%100)	15 (100%)	

MCM, Mini Chromosome Maintenance.

a Statistically significant.

**Table 3:** The relationship between MCMs expression with histopathologic subgrups.

	Histopathologic subgrups			*p*-Value
	Keratinizing	Non-keratinizing	Basaloid	
**MCM2 value**				
Low	1 (6%)	19 (40%)	1 (33%)	0.04^a^
High	15 (94%)	29 (60%)	2 (67%)	
**MCM3 value**				
Low	10 (63%)	30 (63%)	0 (0%)	0.09
High	6 (37%)	18 (37%)	3 (100%)	
**MCM5 value**				
Low	9 (56%)	30 (63%)	0 (0%)	0.1
High	7 (44%)	18 (37%)	3 (100%)	
**MCM6 value**				
Low	4 (25%)	26 (54)	0 (0%)	0.03^a^
High	12 (75%)	22 (46)	3 (100%)	
**MCM7 value**				
Low	6 (38%)	36 (75%)	0 (0%)	0.002^a^
High	10 (62%)	12 (25%)	3 (100%)	

MCM, Mini Chromosome Maintenance.

a Statistically significant.

### Survival outcomes

At a median followup time of 39.7 (range : 6.4–130.8) months, 28 (42%) patients died, 14 (21%) patients had distant metastases and 18 (27%) patients had locoregional progression. During the followup period, 53 (79%) patients had complete response after treatments. The median OS was 75.4 (range : 28.6–122.2) months. The 2- and 5-year OS were 84.5% and 50%, respectively. According to univariate analysis, AJCC stage (stage I–III vs. stage IV; *p* = 0.004), histopathological subgrups (keratinizing vs. non-keratinizing vs. basaloid; *p* = 0.004), tumor response after treatment (complete response vs. non-complete response; *p* = 0.01), MCM2 expression (low vs. High : *p* = 0.02), MCM3 expression (low vs. High : *p* = 0.05), MCM5 expression (low vs. high : *p* = 0.007), MCM6 expression (low vs. High : *p* = 0.04) and MCM7 expression (low vs. high : *p* ≤ 0.0001) were the prognostic factors that predict OS.There were no correlation between survival outcomes and patient gender, age, and chemotherapy administration schedule (concurrent or sequential chemotherapy). According to multivariate Cox regression analysis MCM7 expression was the only prognostic marker for OS. The details of multivariate analyses are shown in Table 4.The median PFS was 59 months. The 2- and 5-year PFS were 85% and 49.5%, respectively. According to univariate analyses, AJCC stage (stage I–III vs. Stage IV : *p* = 0.01), histopathological subgrups (keratinizing vs. non-keratinizing vs. basaloid; *p* = 0.02), tumor response after treatment (complete response vs. non-complete response : *p* = 0.03), MCM3 expression (low vs. high; *p* = 0.04), MCM5 expression (low vs. high; *p* = 0.01), and MCM7 expression (low vs. high; *p* ≤ 0.0001) were the prognostic factors that predict PFS. Similar to OS, MCM7 expression value was the only prognostic factor for PFS according to mutivariate Cox regression analyses.

**Table 4:** Multivariate cox regression analyses related with OS.

Variables	HR	95% CI	*p*-Value
MCM2			
Low	1		
High	1.7	0.54–5.47	0.3
MCM3			
Low	1		
High	1.2	0.49–3.39	0.6
MCM5			
Low	1		
High	2.0	0.73–5.45	0.1
MCM6			
Low	1		
High	2.07	0.55–7.77	0.2
MCM7			
Low	1		
High	2.65	1.01–6.99	0.04^a^
Stage			
I–III	1		
IV	2.03	088–4.71	0.09
Histopathological subgrup			
Keratinizing	1		
Non-keratinizing	0.41	0.15–1.09	0.07
Basaloid	0.58	0.12–2.53	0.45

MCM, Mini Chromoso Memaintenance.

a Statistically significant.

## Discussion

In our study, MCM complex subunits (MCM2–7) overexpression was found as a poor prognostic factor associated with survival outcomes for patients with nasopharyngeal cancer. Although very different results have been presented in the literature with the expression of MCM complex subunits in cancer patients, most of them have shown that MCM complex subunits have an effect on cell proliferation, invasion and metastasis.^19^, ^20^ Liu et al. showed an increase in tumor size and the number of metastatic lymph nodes, progression in the tumor TNM stage, and an increase in tumor invasion rates with MCM2 overexpression in patients with bladder cancer.^21^ Additionally, the rate of recurrence increases with MCM2 overexpression in bladder cancer.^22^ Similarly, Giaginis et al. found that the Dukes stage was more advanced in patients with colorectal cancer with MCM2 overexpression.^23^ It has also been shown that MCM2 overexpression is a poor prognostic factor in glioma, hepatocellular cancers, non-small cell lung cancer, acute lymphocytic leukemia, renal cell cancer and breast cancer.^24^ These results show that biological aggressiveness increases with MCM2 overexpression. In our study, patients with low MCM2 expression demonstrated a significantly better OS than patients with high MCM2 expression. Additionally, patients with low MCM2 expression had better PFS than patients with high MCM2 expression, the results are close to statistical significance. We also showed a statistically significant increase in tumor (T) stage with MCM2 overexpression. Although there is no study in the literature examining the prognostic effect of MCM2 expression in nasopharyngeal cancer, the results of our study are compatible with the literature in that the results are more aggressive in patients with high MCM2 expression.

Compared to other members of MCM complex subunits, the relationship between MCM3 expression and survival has been relatively less studied in the literature. In a previous research, it was showed that the high MCM3 expression enhances cell migration, cell growth, and the invasion ability of medulloblastoma cells.^25^ The poor prognostic effect of MCM3 overexpression was also shown in gliomas, thyroid carcinomas, melanoma, cutaneous T-cell lymphomas and oral squamous cell carcinomas.^19^ In this current study, we have demonstrated that MCM3 overexpression was statistically significantly associated with poor OS and PFS. Although there is no study in the literature examining the prognostic effect of MCM3 expression in nasopharyngeal cancer, these results suggest that MCM3 overexpression may be a prognostic factor for nasopharengeal cancer.

The other parameter investigated in our study was the relationship between MCM5 expression and survival outcomes. According to our study results, the patients with MCM5 overexpression had statistically significant shorter OS and PFS than that of patients with MCM5 low expression. Wang et al. reviewed the role of MCM complex subunits in gastrointestinal malignancies in 2020.^26^ According to their review, MCM complex subunits was seen as prognostic markers for gastrointestinal tumors. They also reported that, MCM5 overexpression correlates with shorter OS in patients with gastric cancer and hepatocellular carcinoma. Nowinska et al. aimed to find the level of MCM5 expression in larengeal squamous cell cancer and compare the results with benign laryngeal lesions.^27^ Their results showed that MCM5 overexpression can be an additional proliferation marker for tumors. The poor prognostic effect of MCM5 overexpression among patients with lung cancer, bladder cancer and hepatocellular cancer were also reviewed by Yu et al. in 2020.^19^ Our results are consistent with the literature in terms of showing the prognostic importance of MCM5 in which overexpression is a significant predictor of poor prognosis.

The prognostic importance of MCM6 overexpression has been verified in various type of cancer like meningiomas, gliomas, hepatocellular cancer, endometrial carcinoma, non-small cell lung cancer, low-grade chondrosarcomas, and mantle cell lymphomas.^19^, ^28^ The authors concluded in these studies that MCM6 overexpression was associated with worse prognosis, similar to our study results. In the current study, patients with high MCM6 expression demonstrated a significantly shorter OS than patients with low MCM6 expression. Additionally, patients with high MCM6 expression had shorter PFS than patients with low MCM6 expression, the results are close to statistical significance.

In univariate analysis, we have demonstrated that MCM7 overexpression was associated with shorter OS and PFS. Also, according to multivariate analysis, high MCM7 expression was the only independent prognostic factor for OS and PFS in the current study. The relationship between cancer and MCM7 expression has been widely examined in the medical literature. Winther and Torp showed that the level of MCM7 protein expression was higher in Grade II meningiomas compared with Grade I meningiomas.^29^ Guan et al. demonstrated that tumor grade increased with elevated MCM7 expression in papillary urothelial cancer.^30^ In esophageal carcinoma cell lines, high MCM7 expression induced cell proliferation, colony formation and migration by regulating the AKT1/mTOR messenger system.^31^ In 2018, Gou et al. evaluated in their meta-analysis based on thirty-one studies investigating the prognostic importance of MCMs in different types of cancer.^20^ As a result, they reported that MCM7 overexpression estimate poor outcomes of patients. Recently, Yu et al. reviewed the significant data related the tumorigenic role of MCM complex subunits and the prognostic importance of MCMs.^19^ As a conclusion, the authors reported that MCM complex subunits are considered as promising prognostic and diagnostic markers, as well as potential therapeutic targets for anticancer treatments.

## Conclusion

Our results have demonstrated that the overexpression of MCM 2, 3, 5, 6 and 7 indicated a poor prognosis. MCM7 was an independent prognostic factor for survival outcomes in nasopharyngeal cancer and may be a potential therapeutic target for treatment. Our findings are consistent with the other study results which demonstrate the prognostic importance of MCM protein family in the other types of cancer.

## Funding

This research did not receive any specific grant from funding agencies in the public, commercial, or not-for-profit sectors.

## Conflicts of interest

The authors declare no conflicts of interest.
